# Vaccination against COVID-19 in the Brazilian indigenous population: Has science been defeated by fake news?

**DOI:** 10.1590/0037-8682-0272-2021

**Published:** 2021-08-20

**Authors:** Marcelo dos Santos Barbosa, Mariana Garcia Croda, Simone Simionatto

**Affiliations:** 1 Universidade Federal de Grande Dourados, Laboratório de Pesquisa em Ciências da Saúde, Dourados, MS, Brasil.; 2 Universidade Federal do Mato Grosso do Sul, Faculdade de Medicina, Campo Grande, MS, Brasil.

Dear Editor:

The COVID-19 pandemic has strongly affected the Brazilian indigenous population, with over 45,000 people infected, an incidence of 5,782 cases/100,000 people and more than 600 deaths^1^. Despite efforts to contain the spread of this disease, the number of cases continues to increase. The second largest indigenous reserve in the country, which is located in the city of Dourados, Mato Grosso do Sul State, saw 445 new cases in January 2021 among its approximately 18,000 indigenous inhabitants-an incidence of 2,472 cases/100,000 inhabitants in 1 month, which is 14% higher than the incidence for the entire year of 2020. Among all indigenous people, 753 were diagnosed with COVID-19 in the first trimester of 2021 alone^1^. These findings indicate that massive vaccination of this population is needed to control the disease. 

**FIGURE 1:**
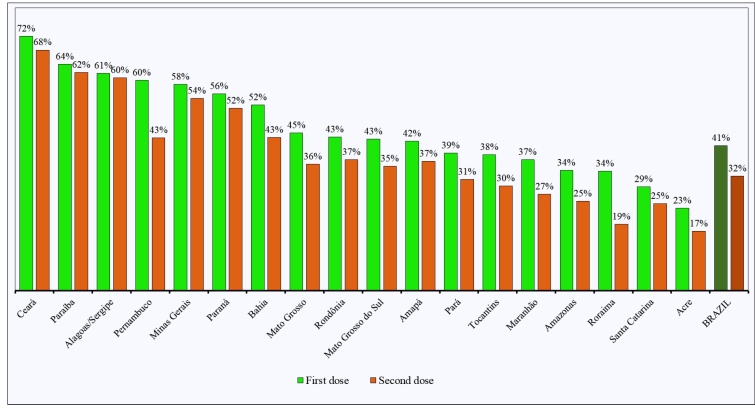
Percentage of COVID-19 vaccine doses in the total Brazilian Indigenous population of each Indigenous Special Sanitary District federative headquarters until April 28, 2021.

In mid-January 2021, an immunization campaign began among indigenous people over 18 years old-approximately 410,000 people. A comprehensive search of the number of indigenous people vaccinated per unit of the federation (UF) was performed using the vaccination monitoring system of the Brazilian health system. By April 28, only 32.44% of the total indigenous population (249,291/774,024 people) and 60.91% of the adult population (249,291/409,883 people) had received the booster dose (Figure 1 and Table 1)^2^. Of the 18 states evaluated, six had not vaccinated more than 30% of the total population with the booster dose, and only two states-Ceará (68%) and Paraíba (62%)-had achieved a 60% vaccination rate among the total indigenous population (Figure 1 and Table 1). 

**TABLE 1:** Percentage of indigenous adults vaccinated using both doses before April 28, 2021.

UF headquarters ISSD	Population		Vaccine in adults			
	Total	Adults	First dose	%	Second dose	%
Ceará	26,129	20,427	18,789	91,98	17,782	87,05
Paraíba	14,024	9,931	8,976	90,38	8,662	87,22
Alagoas/Sergipe	12,250	8,029	7,526	93,74	7,375	91,85
Pernambuco	39,231	26,020	23,364	89,79	16,956	65,17
Minas Gerais	16,684	10,230	9,742	95,23	9,070	88,66
Paraná	22,975	14,481	12,789	88,32	11,85	81,83
Bahia	29,284	20,259	15,365	75,84	12,693	62,65
Mato Grosso	41,120	26,326	18,342	69,67	14,704	55,85
Rondônia	17,470	8,716	7,582	86,99	6,476	74,30
Mato Grosso do Sul	83,434	45,693	35,790	78,33	29,339	64,21
Amapá	12,440	7,288	5,259	72,16	4,551	62,45
Pará	36,405	22,223	14,179	63,80	11,440	51,48
Tocantins	11,908	6,000	4,582	76,37	3,529	58,82
Maranhão	36,060	18,708	13,375	71,49	9,822	52,50
Amazonas	196,054	93,401	66,603	71,31	49,517	53,02
Roraima	78,699	36,072	26,581	73,69	14,790	41,00
Santa Catarina	63,118	20,922	18,551	88,67	15,512	74,14
Acre	31,177	15,157	7,273	47,98	5,223	34,46
**BRAZIL**	**768,462**	**409,883**	**314,668**	**76,77**	**249,291**	**60,82**

UF: Federative Units; ISSD: Indigenous Special Sanitary District.

The Indigenous Special Sanitary District (ISSD) of Mato Grosso do Sul hosts the second largest indigenous population in the country (83,434 people) (Table 1), and it features villages that are geographically accessible to an urban center^3^. For these reasons, several vaccination sites were created inside this ISSD and, in specific cases, health teams were dispatched to patients’ homes. Unfortunately, even with these strategies, the percentage of vaccinated people 2 months later was still below that expected. Prior to April 28, only 35,790 indigenous people had received the first dose of the CoronaVac™ vaccine in ISSD/MS, which corresponds to 43.3% of the total population and 78% of adults. Similarly, 29,613 people had received the booster dose (35.6% of the total population and 64% of adults) (Figure 1 and Table 1)^2^. Because the vaccination uptake was low, the unused doses were distributed to other groups of non-indigenous individuals, such as health professionals and the elderly. 

In 2019, before the coronavirus pandemic and the flood of anti-vaccine news, only three states had an influenza vaccination coverage below 90% among the indigenous population (Amapá: 88.93%, Paraíba: 89.31%, and Mato Grosso do Sul: 82%)^4^. On the other hand, these states had lower rates of vaccination for COVID-19. In addition, the rates of vaccination in Acre, Roraima, and Santa Catarina states, did not reach 30% of the total indigenous population (Figure 1 and Table 1)^2^. The low vaccination uptake among indigenous populations is a public health emergency as it may lead to an increase in the number of cases, especially cases of the new Brazilian SARS-CoV-2 Gamma variant^5^.

The COVID-19 pandemic has exposed the constant social vulnerability of indigenous peoples in Brazil, such as the precariousness of health services, housing conditions, and education^6^ ^,^ ^7^. This scenario, along with fake news about vaccines, must have contributed to the low vaccine roll-out^8^ ^,^ ^9^. Many fake news attempts to disparage the vaccine have made false assertions about its effects, claiming that the vaccine contains monitoring chips or that it leads to genetic alterations^10^. The fake news spread on social media, which is the main source of information for indigenous people, has been buttressed by discourse from important Brazilian politicians^11^. While most world leaders gathered to discuss the vaccine, Brazil, which was once a world example with its National Vaccination Program^8^, saw its leaders discussing and instigating the use of drugs without scientific evidence^11^ ^,^ ^12^.

Unfortunately, the anti-vaccine rhetoric and misinformation circulated throughout the country also reached Brazilian indigenous reserves, thus discouraging vaccination^10^. In addition, some indigenous deaths at the beginning of the vaccination process caused insecurity within this population, even though the incidents were not associated with SARS-CoV-2 vaccination^6^. These factors, in addition to the ever-present lack of educational material produced in indigenous languages, may have contributed to the low vaccination coverage. In a joint effort, universities and some non-governmental organizations have been producing educational material in native indigenous languages since the beginning of the pandemic, trying to clear doubts about COVID-19^6^. These efforts need to be encouraged by the federal government and health agencies to broaden the amount of correct information about this disease and effective ways to reduce its spread. Higher vaccine uptake, extensive testing, use of masks, social isolation, and hand hygiene need to be intensified to reduce the number of new deaths due to COVID-19 among Brazilian indigenous populations.
